# Score for Predicting Active Cancer in Patients with Ischemic Stroke: A Retrospective Study

**DOI:** 10.1155/2021/5585206

**Published:** 2021-05-25

**Authors:** Jiwei Jiang, Xiuli Shang, Jinming Zhao, Meihui Cao, Jirui Wang, Runzhi Li, Yanli Wang, Jun Xu

**Affiliations:** ^1^Department of Neurology, Beijing Tiantan Hospital, Capital Medical University, Beijing, China; ^2^Department of Neurology, The First Affiliated Hospital, China Medical University, Shenyang, China; ^3^Department of Pathology, The First Affiliated Hospital and College of Basic Medical Sciences, China Medical University, China; ^4^Department of Oncology, Cancer Hospital of China Medical University, Liaoning Cancer Hospital & Institute, China

## Abstract

**Background:**

We aimed to examine the differences of clinical characteristics between patients with ischemic stroke with active cancer and those without cancer to develop a clinical score for predicting the presence of occult cancer in patients with ischemic stroke.

**Methods:**

This retrospective study enrolled consecutive adult patients with acute ischemic stroke who were admitted to our department between December 2017 and January 2019. The demographic, clinical, laboratory, and neuroimaging characteristics were compared between patients with ischemic stroke with active cancer and those without cancer. Multivariate analysis was performed to identify independent factors associated with active cancer. Subsequently, a predictive score was developed using the areas under the receiver operating characteristic curves based on these independent factors. Finally, Bayesian decision theory was applied to calculate the posterior probability of active cancer for finding the best scoring system.

**Results:**

Fifty-three (6.63%) of 799 patients with ischemic stroke had active cancer. The absence of a history of hyperlipidemia (odds ratio (OR) = 0.17, 95% confidence interval (CI): 0.06–0.48, *P* < 0.01), elevated serum fibrinogen (OR = 1.72, 95% CI: 1.33–2.22, *P* < 0.01) and D-dimer levels (OR = 1.43, 95% CI: 1.24–1.64, *P* <0.01), and stroke of undetermined etiology (OR = 22.87, 95% CI: 9.91–52.78, *P* < 0.01) were independently associated with active cancer. A clinical score based on the absence of hyperlipidemia, serum fibrinogen level of ≥4.00 g/L, and D-dimer level of ≥2.00 *μ*g/mL predicted active cancer with an area under the curve of 0.83 (95% CI: 0.77–0.89, *P* < 0.01). The probability of active cancer was 59% at a supposed prevalence of 6.63%, if all three independent factors were present in a patient with ischemic stroke.

**Conclusions:**

We devised a clinical score to predict active cancer in patients with ischemic stroke based on the absence of a history of hyperlipidemia and elevated serum D-dimer and fibrinogen levels. The use of this score may allow for early intervention. Further research is needed to confirm the implementation of this score in clinical settings.

## 1. Introduction

Malignant tumors and stroke are the most common causes of disability and mortality worldwide [1]. The concomitance of both conditions has serious repercussions on the quality of life, with a substantial increase in the socioeconomic burden at both the individual and societal levels. Previous studies have reported that approximately 15% of patients with cancer are at risk of developing ischemic stroke (IS) later in life [2], and 10% of patients hospitalized with IS could have cancer as a comorbidity [3]. Sometimes, IS could even represent the initial manifestation of occult cancer [4]. A systematic review revealed that the short-term risk of stroke was considerably increased after a new diagnosis of a solid tumor, and correspondingly, the probability of diagnosing cancer was significantly high in the first few months after a stroke [5]; moreover, the risk of stroke varies with the type of cancer, histopathological features, and stage [6]. This evidence demonstrates a close association between cancer and stroke, revealing an opportunity for occult cancer screening in specific populations, such as patients with cryptogenic stroke. Thus, discerning occult malignancies at an early stage in patients with IS and treatment with appropriate interventions could help improve the chances of survival and functional outcomes [7].

However, there is no consensus on the characteristics of patients with IS at a higher risk of developing malignancies, despite the increasing interest in the relationship between IS and cancer [6, 8]. Some studies have shown that higher rates of stroke of undetermined etiology (SUE), elevated C-reactive protein (CRP) and D-dimer levels, and lesions involving multiple vascular territories as shown on diffusion-weighted imaging (DWI) are relatively specific characteristics of patients with cancer-associated IS [9–11]. However, other studies did not find significant differences in these stroke-related characteristics between IS patients with and without cancer [12–14]. Currently, research focusing on the appropriate time and approach for screening cancer in patients with IS is lacking. To the best of our knowledge, only one study has reported a score for predicting occult malignancies in patients with IS; however, in terms of overall predictive strength, that score had an area under the curve (AUC) of 0.73 [15]. Therefore, we conducted a retrospective study to identify the differences in clinical, laboratory, and neuroimaging characteristics between IS patients with active cancer or without cancer and to develop a more reliable, clinically relevant predictive score for cancer screening in patients with IS.

## 2. Materials and Methods

### 2.1. Patient Selection and Data Collection

This retrospective study enrolled consecutive patients with acute IS from the Department of Neurology at the First Affiliated Hospital of China Medical University, a comprehensive academic hospital in a large urban area, between December 2017 and January 2019. The inclusion criteria were as follows: (1) age ≥ 18 years, (2) IS that met the Baltimore-Washington Cooperative Young Study Criteria, (3) neurologic deficit lasting longer than 24 h, and (4) computed tomography (CT) or magnetic resonance imaging (MRI) scans depicting infarctions consistent with clinical findings [16]. Patients were excluded from the study if they (1) lacked information regarding the etiological examination for stroke, including data from Holter electrocardiography (ECG), transthoracic or transesophageal echocardiography, magnetic resonance angiography (MRA), CT angiography (CTA), or Doppler ultrasonography; (2) were diagnosed with transient ischemic attack (TIA) or cerebral hemorrhage; (3) had a previous history of brain tumor, cerebral metastases, or intracranial surgery; and (4) had indications of inactive cancer (patients with IS and cancer that did not meet the description of active cancer) or hematological malignancies. Active cancer was defined as the diagnosis of cancer or administration of cancer treatment within the past 6 months or the metastasis or recurrence of known cancers [17, 18]. Patients with IS and active cancer were categorized into the cancer group, while those without cancer were assigned to the control group.

### 2.2. Clinical Assessment

Trained personnel reviewed the patients' demographic data (age, sex, and medical history); vascular risk factors (hypertension: previous diagnosis and treatment or blood pressure ≥ 140/90 mmHg); diabetes mellitus (previous diagnosis and treatment or fasting glucose level of ≥7.00 mmol/L); hyperlipidemia (previous diagnosis and treatment; fasting total serum cholesterol level of ≥5.72 mmol/L, triglyceride level of ≥1.7 mmol/L, or low-density lipoprotein level of ≥3.64 mmol/L); atrial fibrillation (previous diagnosis and treatment or suggestive ECG findings); history of coronary heart disease, IS, or TIA; obesity (body mass index ≥ 28 kg/m^2^); current smoking habits; stroke severity on admission (based on the National Institute of Health Stroke Scale (NIHSS) score) [19]; and results of diagnostic investigations. Two board-certified neurologists consensually determined the stroke etiology as large-artery atherosclerosis (LAA), cardioembolism (CE), small vessel occlusion (SVO), stroke of other determined etiology (SOE), or SUE, according to the Trial of Org 10172 in Acute Stroke Treatment (TOAST) criteria [20].

Routine investigations included neuroimaging (MRI, especially contrast-enhanced MRI and DWI, and angiography, including MRA, CTA, and Doppler ultrasonography), ECG or 24 h ECG, and transthoracic or transesophageal echocardiography. The presence of acute multiple cerebral infarctions (AMCI) was determined based on DWI findings, and AMCI were defined as multiple acute infarcts, either in the bilateral anterior or posterior circulation or simultaneously in the anterior and posterior circulations, suggesting an embolic etiology. Data on routine laboratory parameters, including serum platelet counts and hemoglobin, fibrinogen, D-dimer, CRP, lipid, and glucose levels, were also obtained. The diagnosis of cancer was confirmed based on histopathological evidence and the oncologist's opinion. Furthermore, oncological records were carefully reviewed for cancer-specific details, including tumor type, histology, and stage.

### 2.3. Statistical Analysis

All statistical analyses were performed using SPSS 22.0 statistical software (SPSS Inc., Chicago, IL, USA). Categorical variables are presented as the total number (*n*) and percentage (%) per group, and the *χ*^2^ or Fisher's exact test was used to assess statistical differences. The mean and standard deviations (SDs) were calculated for continuous variables with normal distribution, while the median and interquartile range (IQR) were used for continuous variables lacking normal distribution. Similarly, the Student *t*-test was used for normally distributed data. The Mann–Whitney *U* test was used for data without normal distribution. Risk factors (*P* ≤ 0.05) were further analyzed using univariate and multivariate logistic regression. Risk factors independently associated with active cancer were included in our clinical scoring system, which were dichotomized using cut-off points prior to score entry based on clinical experience and previously published evidence. We compared the diagnostic performance of different scores using the area under the receiver operating characteristic (AUC-ROC) curve. Bayesian decision theory was used to calculate the posterior probability of active cancer for different scores. All *P* values were two-tailed. *P* values < 0.05 were considered statistically significant.

## 3. Results

### 3.1. Baseline Characteristics of Patients with Ischemic Stroke in Both Groups

**Figure 1:**
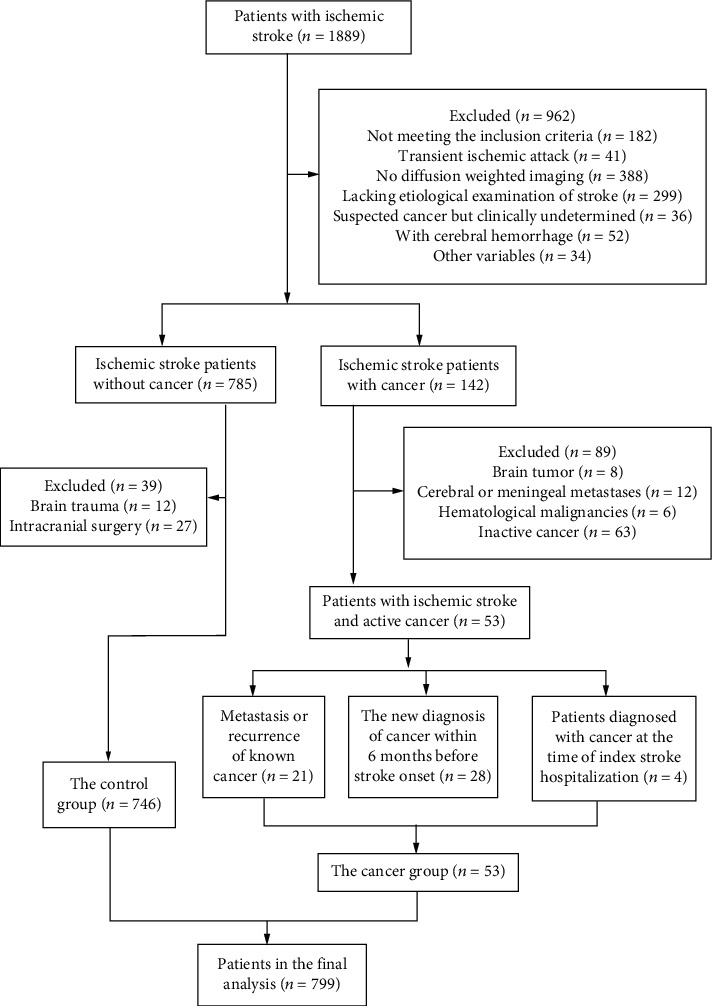
Flow diagram explaining the inclusion and exclusion criteria.

Of the initial 1,889 patients with IS, 799 were included in the final analysis (Figure 1). Of these, 53 (6.63%) were diagnosed with active cancer at the time of stroke onset and were assigned to the cancer group, while 746 (93.37%) did not have a history of cancer and were assigned to the control group.

Table 1 shows the patient characteristics for both groups. Patients in the cancer group had a significantly higher mean age than those without cancer (mean ± SD: 67.21 ± 10.11 years vs. 62.11 ± 12.09 years; *t* = 3.00, *P* < 0.01). The frequency of hyperlipidemia was lower in the cancer group than in the control group (22.6% vs. 49.3%; *χ*^2^ = 14.13, *P* < 0.01). The prevalence of other vascular risk factors did not differ significantly between the two groups. We observed that 35 (66.04%) patients in the cancer group had SUE compared to 31 (4.16%) patients in the control group (*χ*^2^ = 250.06, *P* < 0.01), while the LAA subtype was less common in the cancer group than in the control group (9.43% vs. 60.99%; *χ*^2^ = 53.85, *P* < 0.01), as per the TOAST criteria. There were no significant differences in prevalence of the CE, SVO, and SOE subtypes. We found that the average NIHSS score at admission was higher in the cancer group than in the control group (mean ± SD: 6.13 ± 4.10 vs. 4.30 ± 4.48; *t* = 2.89, *P* < 0.01). AMCI were observed more frequently in the cancer group than in the control group (56.60% vs. 19.84%; *χ*^2^ = 38.63, *P* < 0.01). The incidence of deep vein thrombosis or pulmonary embolism during hospitalization was higher in the cancer group than in the control group (13.21% vs. 3.75%; *χ*^2^ = 10.56, *P* < 0.01). Patients with active cancer had significantly higher serum levels of fibrinogen (mean ± SD: 4.44 ± 1.82 g/L vs. 3.56 ± 1.02 g/L; *t* = 3.49, *P* < 0.01), D-dimer (median [IQR]: 2.23 mg/mL [0.98–10.76] vs. 0.35 mg/mL [0.27–0.48]; *Z* = −9.55, *P* < 0.01), and CRP (median [IQR]: 18.40 mg/L [5.45–46.00] vs. 3.90 mg/L [2.80–6.33]; *Z* = −7.35, *P* < 0.01) but significantly lower levels of hemoglobin (mean ± SD: 119.79 ± 25.68 g/L vs. 143.61 ± 18.57 g/L; *t* = −6.63, *P* < 0.01) than those without.

### 3.2. Type, Histology, and Stage of Active Cancer

The most commonly observed cancer diagnoses among patients with IS and active cancer were as follows: lung cancer (*n* = 16, 30.19%); gastric cancer (*n* = 8, 15.09%); liver cancer (*n* = 6, 11.32%); colorectal cancer (*n* = 5, 9.43%); breast cancer (*n* = 5, 9.43%); genitourinary cancers (bladder, prostate, and ovarian cancers; *n* = 5, 9.43%); biliary tract cancer (*n* = 4, 7.55%); and pancreatic cancer, nasopharyngeal cancer, renal cancer, and adrenal cancer (*n* = 1, 1.89% each). Thirty-one (58.49%) patients were diagnosed with adenocarcinoma, and 21 (39.62%) were diagnosed with metastatic disease (Table 2).

### 3.3. Univariate and Multivariate Logistic Regression Analyses

We assessed all risk factors with *P* values ≤ 0.05 (Table 1) using a univariate logistic regression model (including age, hyperlipidemia, LAA, SUE, NIHSS score, AMCI, hemoglobin, fibrinogen, D-dimer, and CRP). The results are shown in Table 3. Notably, the odds ratios (OR) for age, NIHSS score, hemoglobin, and CRP were approximately 1 on univariate logistic regression analysis. Therefore, we considered only the remaining risk factors, including hyperlipidemia, SUE, AMCI, presence of venous thromboembolism or pulmonary embolism, and fibrinogen and D-dimer levels, in the multivariate logistic regression model. Multivariate analysis showed that the absence of a history of hyperlipidemia (OR = 0.17, 95% confidence interval (CI): 0.06–0.48, *P* < 0.01), elevated serum fibrinogen levels (OR = 1.72, 95% CI: 1.33–2.22, *P* < 0.01), elevated D-dimer levels (OR = 1.43, 95% CI 1.24–1.64, *P* < 0.01), and SUE (OR = 22.87, 95% CI: 9.91–52.78, *P* < 0.01) were independently associated with active cancer (Table 3).

### 3.4. Development of a Predictive Score Using Areas under the Receiver Operating Characteristic Curves

**Figure 2:**
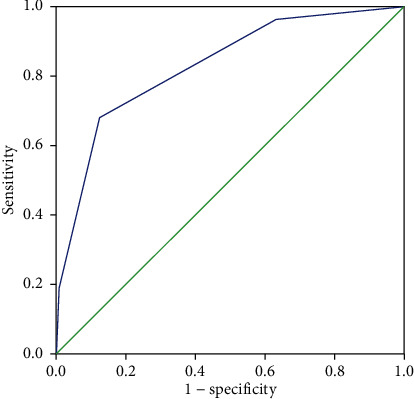
Receiver operating characteristic curves of the clinical scores. The final scores were as follows: history of hyperlipidemia = 0 points, no history of hyperlipidemia = 1 point; D-dimer level ≤ 2.00 *μ*g/mL = 0 points, D-dimer level > 2.00 *μ*g/mL = 1 point; and fibrinogen level ≤ 4.00 g/L = 0 points, fibrinogen level > 4.00 g/L = 1 point.

We developed a scoring system to predict active cancer in patients with IS, based on the findings of multivariate analysis, particularly in the subset of patients with SUE. The final score, ranging from 0 to 3, comprised the sum of individual scores for the history of hyperlipidemia, serum D-dimer levels, and serum fibrinogen levels. We reviewed and compared several studies to determine the appropriate cut-off values for D-dimer and fibrinogen levels for the scoring system. The OASIS-CANCER study conducted by Lee et al. determined the first quartile of the pretreatment D-dimer concentration as 2.08 *μ*g/mL and the median fibrinogen concentration as 399 mg/dL [21]. Moreover, Quintas et al. found that the median fibrinogen level of patients with IS without cancer was 408.5 mg/dL [22]. They demonstrated that fibrinogen values were associated with the diagnosis of cancer after IS. Therefore, we analyzed multiple data conditions and assigned the following final scores, according to the findings of previous studies, our clinical experience, and the findings of this study: history of hyperlipidemia = 0 points, no history of hyperlipidemia = 1 point; D-dimer level ≤ 2.00 *μ*g/mL = 0 points, D-dimer level > 2.00 *μ*g/mL = 1 point; and fibrinogen level ≤ 4.00 g/L = 0 points, fibrinogen level > 4.00 g/L = 1 point.  Table 4 presents the sensitivity, specificity, and posterior probability of each cut-off point based on the supposed cancer prevalence value of 6.63% in our study.  Figure 2 shows that the probability of active cancer was 59% if a patient with IS had a clinical score of 3 points, with a reliable AUC-ROC value of 0.83 (95% CI: 0.77–0.89, *P* < 0.01).

## 4. Discussion

This study showed that the absence of a history of hyperlipidemia, increased serum levels of D-dimer and fibrinogen, and SUE were independent risk factors associated with active cancer in patients with IS. Adenocarcinoma was the most commonly observed histological manifestation, consistent with the results of several previous studies [21, 23, 24]. However, recent studies evaluating underlying cancer in patients with IS have simply focused on differences in epidemiological and biochemical parameters. There is no consensus on the optimal method or time for identifying occult cancer in patients with acute IS. Thus, in light of our findings, we developed a reliable predictive score that can help clinical neurologists to rapidly screen the possibility of occult cancer in patients with IS at an early stage, which could ultimately improve patients' quality of life and survival outcomes.

Our systematically developed predictive score consisted of increased serum levels of D-dimer and fibrinogen and the absence of a history of hyperlipidemia, especially in patients with SUE. Our final scoring system demonstrated that if a patient with IS had a total score of 3/3, the probability of active cancer was 59% (with high specificity [99%]), based on an assumed cancer prevalence of 6.63%, which was similar to that in previous studies [22, 25, 26]. The probability of active cancer was 27% if the score was 2 points. We compared the predictive capabilities of our scoring system with those of a previously reported system and found that our system was significantly superior; the AUC of the score described by Selvik et al. was 0.73 [15], while the AUC of our score was 0.83.

The differences in classical vascular risk factors between patients with IS with and without cancer remain unclear. Some studies have reported that the prevalence of classical vascular risk factors was similar between these patient groups [18, 27, 28], while others have demonstrated that the frequency of vascular risk factors, especially hyperlipidemia, was lower in patients with IS and active cancer [29, 30]. Our analyses demonstrated that the absence of a history of hyperlipidemia was independently associated with the presence of active cancer in patients with IS. Previous studies have suggested that low total cholesterol levels were associated with an elevated risk of cancer incidence and mortality [31–33], and this inverse association has been well demonstrated in patients with stomach, breast, and prostate cancers [32, 34]. The findings of these studies are generally in accordance with those of our study. Moreover, we found a significant difference in the classification of stroke etiology between the two groups. Similar to previous studies, we observed a lower frequency of the LAA subtype and a higher prevalence of SUE in patients with IS and active cancer than in those without cancer [12, 29]. Thus, previous studies and our study indicate that a specific stroke mechanism, differing from that associated with traditional IS, may exist in patients with active cancer.

We compared the differences in blood coagulation biomarkers between the two groups, including an analysis of platelet, fibrinogen, and D-dimer levels to further explore the possible mechanisms underlying cancer-associated IS. We found higher serum levels of fibrinogen and D-dimer in patients with IS and active cancer than in those without cancer, similar to most recent studies [11, 21, 22]. These factors are even considered to be predictors of active cancer in patients with IS [35]. Earlier studies have reported that D-dimer levels, which reflect excessive fibrin turnover associated with an activated coagulation system, were correlated with the degree of hypercoagulability [21] and widespread dissemination of microthrombi [36]. Moreover, malignancies can induce a hypercoagulable state and promote the formation of microthrombi by promoting the secretion of mucins, release of tissue factors, and production of procoagulant cytokines [37], which may explain the elevated levels of blood coagulation biomarkers in the present study. Therefore, it is necessary to identify the specific cancer types that tend to induce hypercoagulable states and thrombosis. Our data demonstrated that active lung cancer and gastric cancer were the most common cancer types in IS patients, and adenocarcinoma was the most commonly observed histopathological subtype. These findings are also consistent with those of previous studies, which revealed a higher prevalence of thromboembolic events in patients with lung cancer and adenocarcinoma [25, 38, 39]. Furthermore, studies have shown that patients with adenocarcinoma were prone to developing cancer-mediated hypercoagulability and microemboli [18, 40]. Several studies have suggested that some epithelium-derived tumors, such as those originating from the lung, stomach, and bile ducts, were frequently adenocarcinomas and could systematically secrete mucins that bind to P- and L-selectins, inducing the formation of platelet-rich microthrombi [41, 42]. Moreover, these cancers usually remain undiagnosed until they reach an advanced or metastatic stage. The histologic and natural characteristics of these tumors underline the potential increase in the risk of IS, further revealing the role of hypercoagulability in cancer-related IS.

Our study has several limitations. First, this was a single-center retrospective study performed using consecutively collected data. Therefore, our findings may not be applicable to other settings due to the inherent selection bias. Second, comparing the characteristics of patients according to different cancer types would have been ideal, as patients with IS may have different types of cancer. However, further categorization by cancer type did not allow for appropriate subgroup analyses, because this study included a relatively limited number of patients with active cancer. Third, we could not clarify the relationship between the value of hypercoagulation biomarkers and tumor markers, as this is difficult to achieve for a retrospective study; furthermore, tumor markers were not routinely tested in the Department of Neurology due to Chinese national conditions and the high cost of examination.

## 5. Conclusions

This study systematically developed a scoring system comprising the history of hyperlipidemia and fibrinogen and D-dimer levels for the prediction of active cancer in patients with IS, especially in those with SUE. Our findings indicate the importance of hypercoagulability in assessing active cancer in patients with IS, which could support early decision-making for intervention and management at the time of admission. Prospective multicenter studies are needed to evaluate the effectiveness of this clinical scoring system prior to implementation in clinical practice.

## Figures and Tables

**Table 1:** Comparison of baseline characteristics between patients with ischemic stroke and active cancer and those without cancer.

Variables	Cancer group *n* = 53	Control group *n* = 746	*t*/*χ*^2^	*P* value
*Demographics*				
Age (years)	67.21 ± 10.11	62.11 ± 12.09	3.00	<0.01
Sex (% male)	34 (64.2)	522 (70.0)	0.79	0.37
Vascular risk factors				
Hypertension	30 (56.6)	508 (68.1)	2.97	0.09
Diabetes mellitus	14 (26.4)	286 (38.3)	3.00	0.08
Hyperlipidemia	12 (22.6)	368 (49.3)	14.13	<0.01
Atrial fibrillation	4 (7.6)	46 (6.2)		0.57
Coronary heart disease	8 (15.1)	106 (14.2)	0.03	0.86
Previous stroke/TIA	7 (13.2)	129 (17.3)	0.59	0.45
Current smoking	25 (47.2)	351 (47.1)	0.00	0.99
Obesity	6 (11.3)	121 (16.2)	0.89	0.35
*TOAST classification*				
Large-artery atherosclerosis	5 (9.4)	455 (61.0)	53.85	<0.01
Cardioembolism	2 (3.8)	33 (4.4)		1.00
Small vessel occlusion	9 (17.0)	216 (29.0)	3.51	0.06
Other determined etiology	2 (3.8)	11 (1.5)		0.21
Undetermined etiology	35 (66.0)	31 (4.2)	250.6	<0.01
*Ischemic stroke characteristics*				
Initial NIHSS score	6.13 ± 4.10	4.30 ± 4.48	2.89	<0.01
AMCI	30 (56.6)	148 (19.8)	38.63	<0.01
VTE or PE	7 (13.2)	28 (3.8)	10.56	<0.01
*Laboratory markers*				
Hemoglobin (g/L)	119.79 ± 25.68	143.61 ± 18.58	-6.63	<0.01
Platelets (/10^9^)	213.28 ± 99.86	222.17 ± 66.55	-0.64	0.53
Fibrinogen (g/L)	4.44 ± 1.82	3.56 ± 1.02	3.49	<0.01
D-dimer (mg/mL)	2.23 (0.98–10.76)	0.35 (0.27–0.48)	-9.55	<0.01
CRP (mg/L)	18.40 (5.45–46.00)	3.90 (2.80–6.33)	-7.35	<0.01

Data are presented as the mean ± standard deviation, median (interquartile range), or *n* (%). Abbreviations: AMCI: acute multiple cerebral infarctions; VTE: venous thromboembolism; PE: pulmonary embolism; CRP: C-reactive protein; NIHSS: National Institute of Health Stroke Scale; TOAST: Trial of Org 10172 in Acute Stroke Treatment; TIA: transient ischemic attack.

**Table 2:** Characteristics of patients with ischemic stroke and active cancer.

Cancer location	Histological type	Number
Lung cancer	Adenocarcinoma	15
	Small cell carcinoma	1
Gastric cancer	Adenocarcinoma	8
Liver cancer	Hepatocellular carcinoma	6
Colon cancer	Adenocarcinoma	3
Rectal cancer	Adenocarcinoma	2
Breast cancer	Infiltrating ductal carcinoma	4
	Medullary carcinoma	1
Biliary tract cancer	Epithelial cell carcinoma	3
	Adenocarcinoma	1
Bladder cancer	Transitional epithelial carcinoma	3
Prostate cancer	Adenocarcinoma	1
Ovarian cancer	Serous carcinoma	1
Pancreatic cancer	Adenocarcinoma	1
Nasopharyngeal cancer	Squamous cell carcinoma	1
Renal cancer	Clear cell carcinoma	1
Adrenal cancer	Cortical carcinoma	1
Metastatic disease		21

**Table 3:** Univariate and multivariate logistic regression analyses of risk factors in patients with ischemic stroke with active cancer and those without cancer.

Variables	Univariate logistic regression analysis		Multivariate logistic regression analysis	
	OR (95% CI)	*P* value	OR (95% CI)	*P* value
Age	1.04 (1.01–1.07)	<0.01		
Hyperlipidemia	0.30 (0.16–0.58)	<0.01	0.16 (0.06–0.45)	<0.01
LAA	0.07 (0.02–0.17)	<0.01		
SUE	44.85 (22.89–87.88)	<0.01	19.30 (7.93–48.96)	<0.01
NIHSS score	1.06 (1.02–1.11)	<0.01		
AMCI	5.27 (2.97–9.34)	<0.01	1.58 (0.66–3.81)	0.31
VTE or PE	4.56 (1.97–10.58)	<0.01	0.92 (0.21–3.94)	0.91
Hemoglobin (g/L)	0.95 (0.94–0.96)	<0.01		
Fibrinogen (g/L)	1.63 (1.35–2.00)	<0.01	1.70 (1.31–2.21)	<0.01
D-dimer (*μ*g/mL)	1.72 (1.45–2.03)	<0.01	1.42 (1.23–1.65)	<0.01
CRP	1.03 (1.02–1.04)	<0.01		

LAA: large-artery atherosclerosis; SUE: stroke of undetermined etiology; NIHSS: National Institute of Health Stroke Scale; AMCI: acute multiple cerebral infarctions; VTE: venous thromboembolism; PE: pulmonary embolism; CRP: C-reactive protein.

**Table 4:** Performance and posterior probabilities of the predictive score for cancer in patients with ischemic stroke.

Score	Sensitivity	Specificity	Posterior probability
1	0.96	0.37	0.09
2	0.68	0.88	0.27
3	0.19	0.99	0.59

## Data Availability

Data has not been made accessible in the interest of protecting patients' privacy.
